# Congenital Epignathus Associated With a Tricuspid Incompetence

**DOI:** 10.7759/cureus.27135

**Published:** 2022-07-22

**Authors:** Rami Joseph, Omer Abdoun, Esraa M Abdelrahim, Aisha K Babiker, Abeer H Mohammed

**Affiliations:** 1 Oral and Maxillofacial Surgery, Sudan Medical Specialization Board, Khartoum, SDN; 2 Research, Khartoum Teaching Dental Hospital, Khartoum, SDN; 3 Oral and Maxillofacial Surgery, University of Khartoum, Khartoum, SDN; 4 Oral and Maxillofacial Surgery, Khartoum Teaching Dental Hospital, Khartoum, SDN; 5 Oral Pathology, University of Khartoum, Khartoum, SDN

**Keywords:** germinal cell neoplasms, congenital epignathus, embryonic germ layers, palatal swelling, true teratoma

## Abstract

Teratomas are germinal cell neoplasms containing tissues from all germinal layers (ectoderm, mesoderm, and endoderm); however, they may show varying stages of maturity. We present a case of a 10-month-old Sudanese male patient who presented to the Khartoum Teaching Dental Hospital with a tumor occupying the hard palate since birth, causing reduced oral intake and failure to thrive. CT images showed soft and hard tissue masses originating from the hard palate and extending to the oral cavity. The treatment was initiated with nutritional support and consultation with pediatric physicians. The oral teratoma was successfully treated with surgery. The patient was regularly followed up and he fully recovered eventually.

## Introduction

As defined by Weaver et al., teratoma is a tumor composed of multiple tissues and foreign to the part in which it arises [1]. Teratomas typically consist of tissues from all three embryonic germ layers: ectoderm, mesoderm, and endoderm. Grossly, they are heterogeneous masses composed of solid and cystic components [2].

These tumors are histologically benign ordinarily, although they result in significant morbidity and potential mortality due to their size and anatomical site, leading to airway obstruction and respiratory distress [3]. They frequently originate from a midline or paraxial site from the brain to the sacral area and are most often located in the sacrococcygeal region. Of note, 90% of the head and neck teratomas present during the neonatal and infantile periods, mainly involving the neck and nasopharynx. They have an incidence rate of one in 20,000-40,000 births [4].

## Case presentation

**Figure 1:**
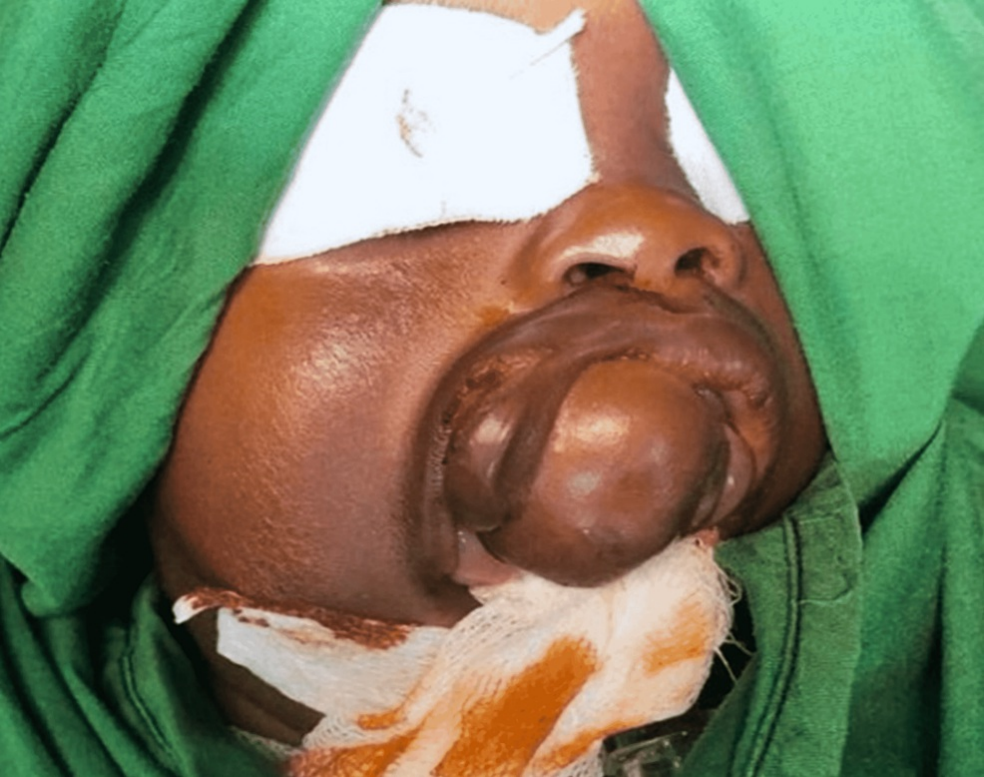
Swelling is seen in the anterior maxilla obstructing the oral cavity

A 10-month-old Sudanese male child who weighed 5 kg presented to the Oral and Maxillofacial Department at the Khartoum Teaching Dental Hospital with his parents, reporting an oral swelling in the hard palate and protruding extra-orally (Figure 1). It had been present since birth with no change in size, and the swelling was causing difficulties in breathing and feeding and had resulted in failure to thrive. There had been no relevant medical events during the pregnancy and delivery.

Head and neck examination

A round swelling was observed in the anterior maxilla obstructing the oral cavity. The swelling was 5 x 2 cm in diameter, with a firm consistency. The overlying skin was intact and of normal color and texture, with some hair follicles seen, and it was not pulsatile or compressible; furthermore, a small soft tissue was seen attached to its posterior end measuring 1.5 x 1.5 in diameter. The swelling extended intraorally and was attached to the mucosa and hard palate in the midline. No teeth were seen in the oral cavity, and there were no other remarkable abnormalities.

Radiograph and investigation

**Figure 2:**
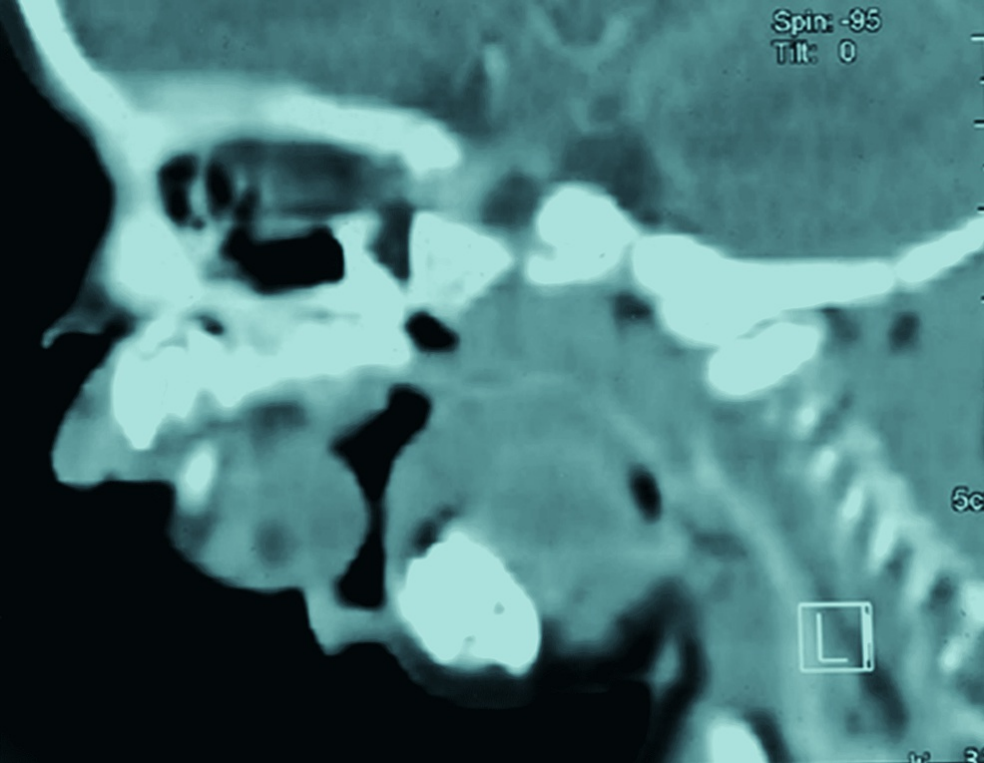
CT scan with contrast: sagittal view showing significant oral tumor CT: computed tomography

**Figure 3:**
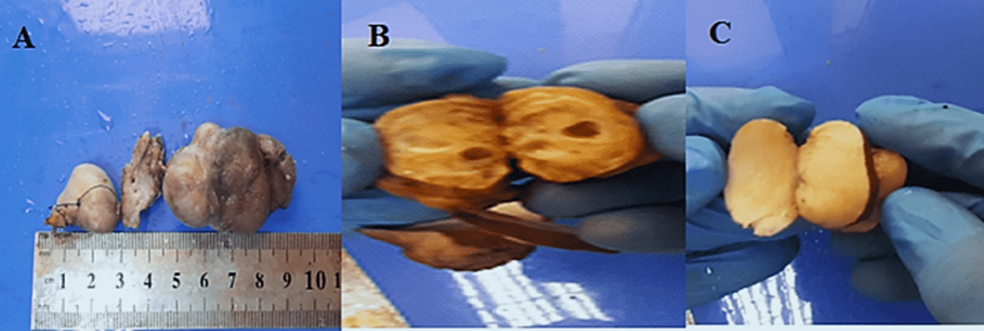
Gross morphology of the mass A: fragments of biopsy. B: cut section shows the cystic area. C: cut section shows the solid area

A complete metabolic panel was performed, which returned normal. In addition, echocardiography showed mild tricuspid regurgitation (TR). A CT head with contrast (Figure 2) showed that the tumor was attached to the hard palate and contained a tooth. The swelling was utterly excised under general anesthesia, and the specimen was sent for a histopathology assessment. The gross morphology of the specimen showed five fragments of formalin-fixed biopsy (two soft tissues and three fragments of hard tissue measuring 9.0 x 2.0 x 1.5 cm collectively). The largest soft tissue measured 5.0 x 2.0 x 1.5 cm, with the hard tissue biopsy measuring 4.0 x 1.5 x 1.0 cm in aggregate left in 20% acid for decalcification for three days. The tumor was encapsulated, and the cut section was firm whitish with solid and cystic areas (Figure 3).

**Figure 4:**
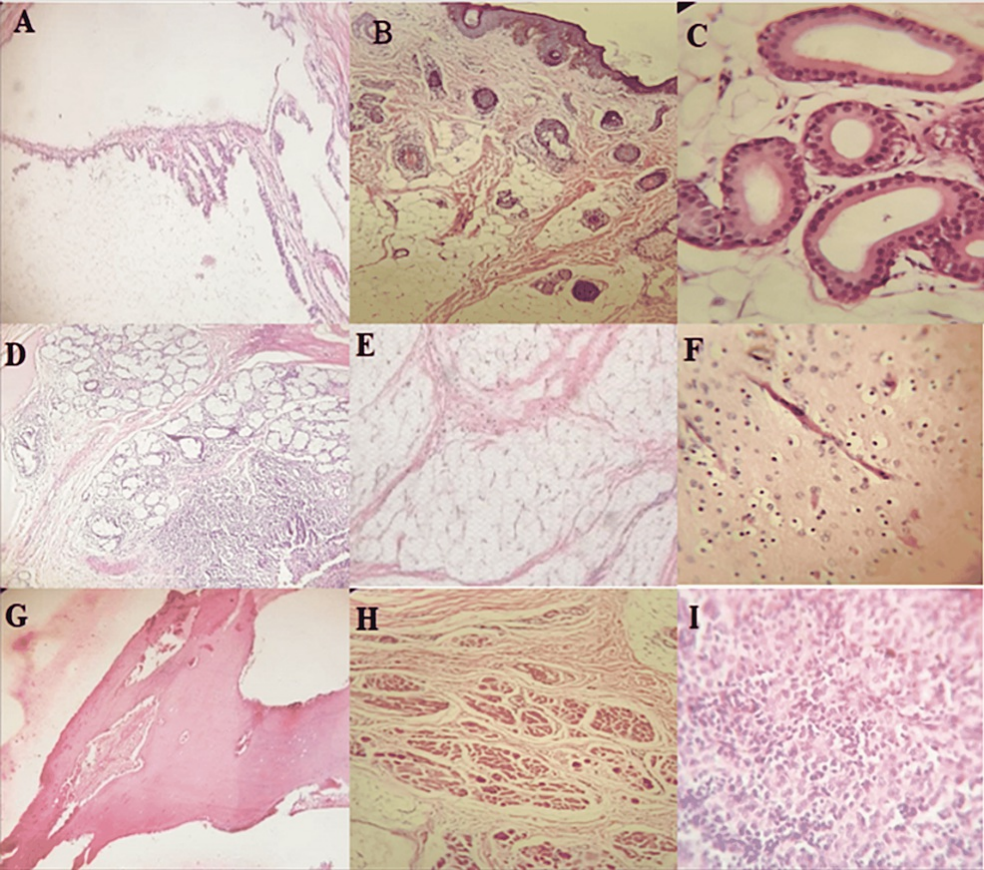
Histopathological results A: section shows solid with cystic areas. B: skin and skin appendages. C: glands. D: salivary glands. E: adipose tissue. F: glial tissue. G: bone trabeculae. H: muscles. I: focus shows the immature area

Histopathological results showed a benign tumor, well-differentiated tissue consisting of glial and adipose tissue, skin appendages muscle, minor salivary gland and sweat gland, and bone trabeculae with the focal area showing undifferentiated tissue (mature teratoma grade I) (Figure 4). A pediatric consult was made, and the team advised multivitamins and a growth formula to improve his weight and hemoglobin levels. In addition, the cardiology team managed the TR conservatively and continued their follow-up postoperatively.

## Discussion

The term "teratoma" comes from the Greek word "teraton" meaning monster and was initially used by Virchowin in 1863 [5]. The histological origin of teratoma continues to be a subject of controversy. The commonly accepted hypothesis proposes that it is likely due to some cellular membrane chemistry modifications; teratomas evolve from displaced totipotent embryonic tissues during early development. Consequently, a collection of tissues is frequently foreign to their araising site [6]. Nevertheless, the exact origin of teratomas is unknown, although many theories have been proposed, including germ cell theory, embryonic cell theory, unifying hypothesis theory, extraembryonic cell theory, twin hypothesis, and the fetus-in-fetus theory [7,8].

The WHO classification of germ cell tumors distinguishes between two separate entities: teratomas with different maturity levels and teratomas with malignant transformation (also called teratoma with somatic-type malignancy). In immature teratomas, primitive neuroectodermal structures predominate. According to the grading system (Gonzalez-Crussi, 1982), mature teratomas (G0) are more frequent (54.5%) than immature teratomas (G1-G3, 45.5%). Only 7.8% of all teratomas show the highest grade of immaturity (G3). The frequency of additional microscopic foci of malignant yolk sac tumors correlates with immaturity grade [9]. In our case, most of the components were mature with a focal area of the immature component, and it was classified as mature teratoma grade 1. Lesions have been classified according to histological components into four types [7]. Firstly, dermoid tumors composed of ectoderm and mesoderm are the most recognizable form of teratoma. Secondly, teratoid tumors have poorly differentiated lesions comprising all germinal layers. Thirdly, true teratomas have identifiable tissue histologically from all germ layers. Lastly, epignathus contains fetal organs. Epignathus is a misleading term that historically means "upon the jaw"; nevertheless, it is applied to nearly all teratomas, whether originating from the oral cavity, pharyngeal tissues, or bulging from the mouth.

While prenatal diagnosis is possible by ultrasonography [10], our patient did not have an antenatal assessment; thus, the mass was detected only after delivery, which had been vaginal in this case.

The most typical finding associated with head and neck teratomas antenatally is polyhydramnios; however, it is mainly observed with large teratomas involving the cervical and nasopharyngeal regions rather than isolated oral teratomas. A baby with suspected congenital oral teratoma should be electively delivered through cesarean section.

**Figure 5:**
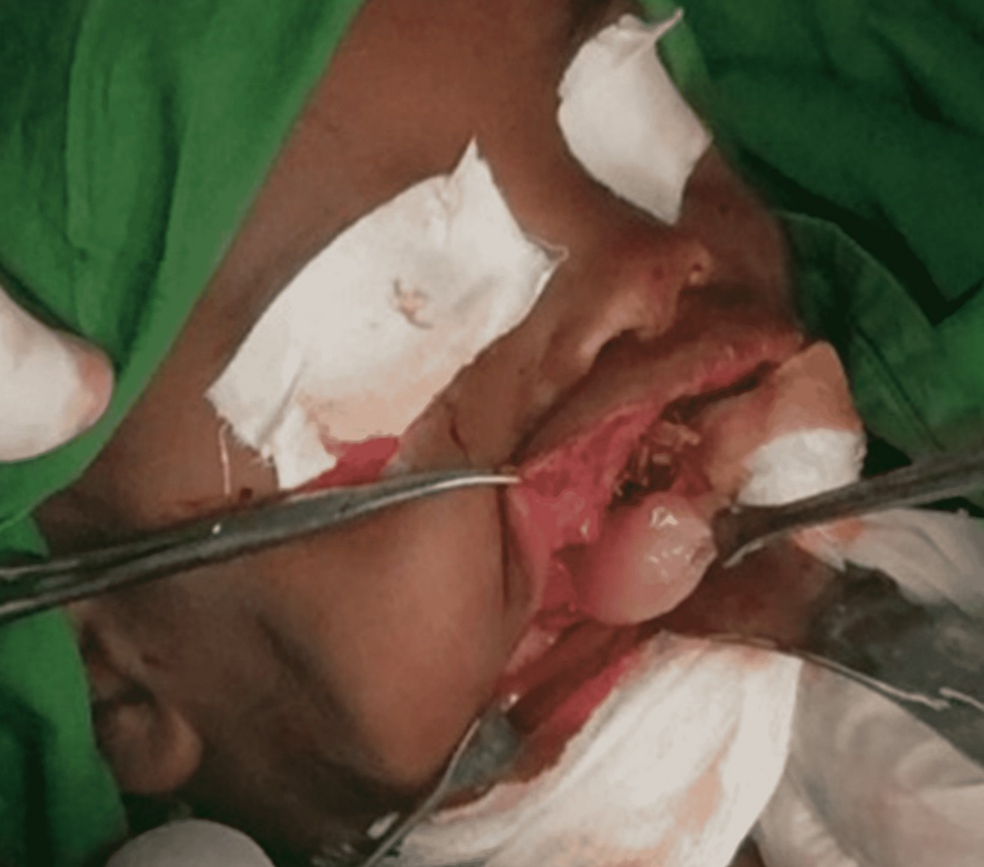
Surgical excision of the tumor

Oral cavity teratomas are predisposed to protrude outside the mouth upon growth rather than posteriorly toward the oropharynx. Given that newborns are necessary nasal breathers, oral obstruction would gradually impact the feeding process rather than causing acute airway compromise. Concomitant anomalies could be associated with oral teratomas, like cystic hygroma, cleft lip/palate, and other multifocal teratomas [3]. Other abnormalities include branchial cleft cyst [11], bifid tongues, bifid nose [12,13], cardiac abnormalities [14], and microcephaly [15]. Heart echocardiography showed mild TR with a normal ejection fraction (69%) in our patient. Furthermore, soft tissue was attached to the mass in the posterio-inferior part and histologically showed the apocrine gland (Figure 5).

**Figure 6:**
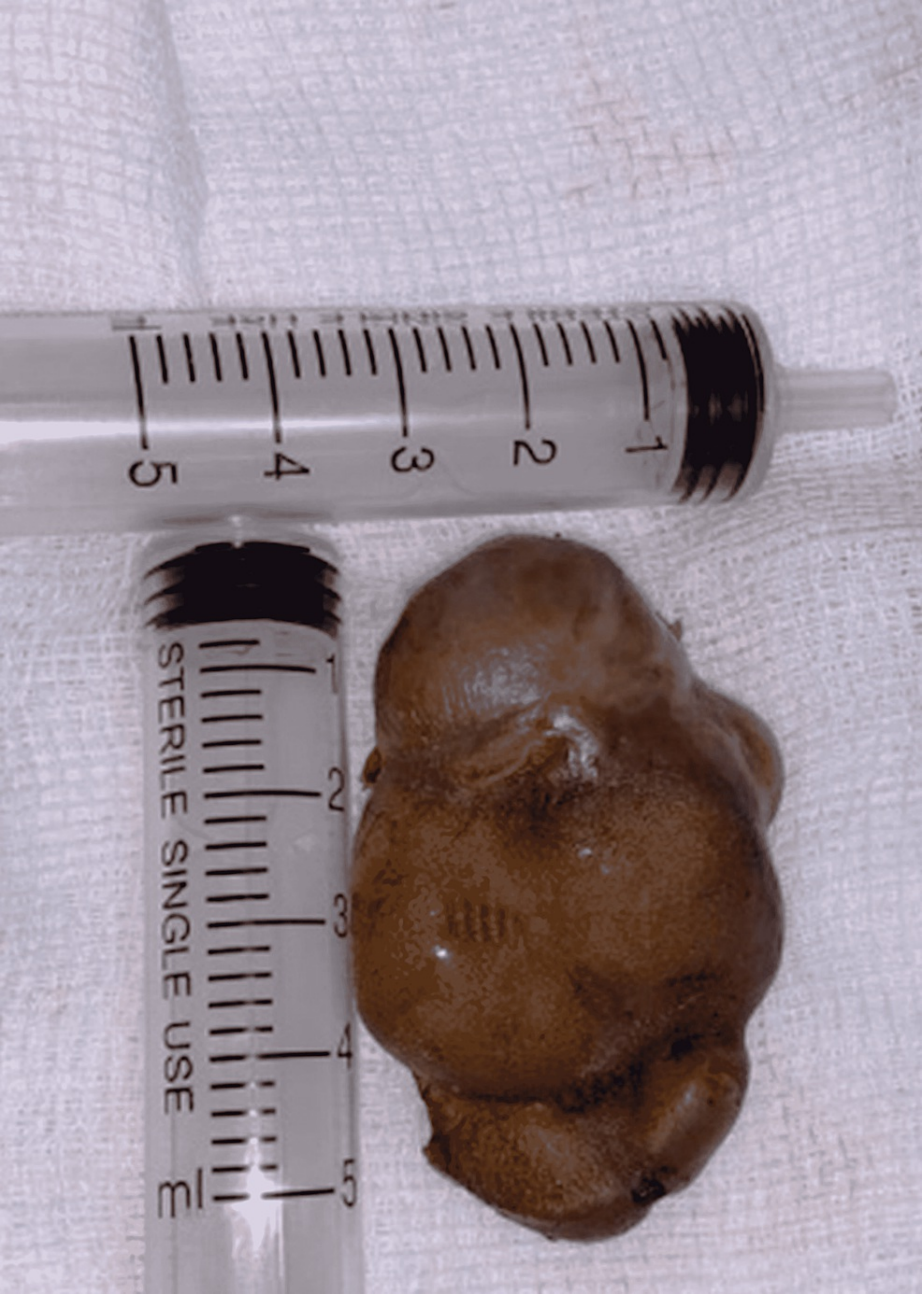
Tumor post excision

**Figure 7:**
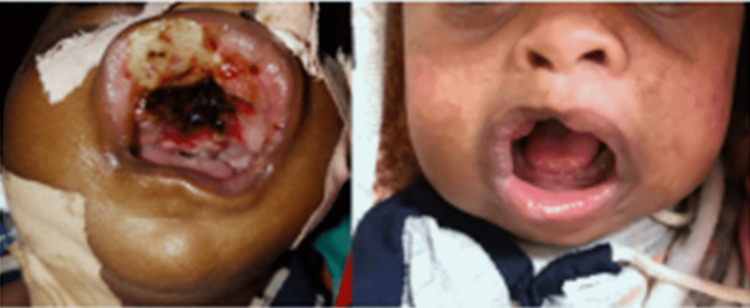
Patient post tumor excision

The primary treatment of teratoma is complete surgical excision, which depends on the tumor site. Unless the teratoma expands massively into the cranial area, the tumor bulk resection may be attempted. Initial treatment should be directed toward airway management and nutrition. Surgical airway and feeding should be considered early [16-18]. In our case, the mass on the palate made oral feeding difficult and had to be surgically removed (Figures 6, 7).

The prognosis for teratoma is excellent. Recurrences are rare and occur predominantly due to incomplete surgical resection [19]. Furthermore, undiagnosed or neglected teratomas involving the head and neck region are associated with a tremendous malignant transformation risk reaching as high as 90%, particularly in adolescence. While the malignant transformation process of oral teratomas is ambiguous, immediate complete resection is mandatory regardless of tumor size or associated complications [9]. To the best of our knowledge, this is the first case report on congenital epignathus from Sudan, and we hope our findings would aid in spreading awareness about such an entity with an excellent prognosis and emphasizing the significance of antenatal care follow-up, thereby reducing the risk of complications during delivery.

## Conclusions

Congenital palatal teratoma is a rare entity with several morphological and histological subtypes, and it is associated with numerous congenital anomalies. However, early intervention with complete resection results in excellent outcomes and substantially low recurrence rates.
